# Ten tips for an onco-nephrology clinic

**DOI:** 10.1093/ckj/sfaf174

**Published:** 2025-05-29

**Authors:** Elena-Bianca Barbir, Nelson Leung, Sandra M Herrmann

**Affiliations:** Department of Internal Medicine, Division of Nephrology and Hypertension, Mayo Clinic, Rochester, MN, USA; Department of Internal Medicine, Division of Nephrology and Transplant, University of Alberta, Edmonton, Canada; Department of Internal Medicine, Division of Nephrology and Hypertension, Mayo Clinic, Rochester, MN, USA; Department of Internal Medicine, Division of Nephrology and Hypertension, Mayo Clinic, Rochester, MN, USA

**Keywords:** acute kidney disease, immune checkpoint inhibitor, immunology, onco-nephrology, thrombotic microangiopathy

## Abstract

An increasing number of cancer patients are benefiting from long term cancer-directed therapy with an ever-expanding arsenal of novel agents from monoclonal antibodies to small molecules and cellular therapies in addition to the mainstay cytotoxic chemotherapy. All these therapies are accompanied by a unique array of adverse events, which include kidney toxicity. In this context, the need for onco-nephrology expertise continues to grow. Oncologists and hematologists collaborate closely with onco-nephrologists to determine: (i) treatment options for special populations based on accurate assessment of patients’ glomerular filtration rate, (ii) supportive therapies for those whose treatment course is complicated by acute kidney injury, and (iii) pharmacologic strategies to continue or restart cancer therapy during kidney dysfunction. Here we outline 10 tips for common clinical scenarios in an outpatient onco-nephrology clinic.

## INTRODUCTION

Onco-nephrology exists as an emerging sub-specialty situated at the intersection among nephrology, oncology, hematology and arguably transplantation and immunology with the related sub-specialty of transplant oncology. Having only surfaced as a specialty ∼15 years ago, it has since grown significantly on an international stage [1]. Its first dedicated journal, the *Journal of Onco-Nephrology* was created in 2017. In 2018 the Kidney Disease: Improving Global Outcomes (KDIGO) group held its first onco-nephrology Controversies Meeting and in 2020 the European Renal Association/European Dialysis and Transplantation Association offered its first symposium on onco-nephrology. In 2021, the Spanish Society of Nephrology created an onco-nephrology working group, and in 2022 the American Society of Onco-Nephrology was created [1, 2]. The demand for onco-nephrology expertise will only continue to increase as (i) more patients fit into the modern paradigm of cancer as chronic disease, in which patients benefit from long term cancer-directed therapy to prevent progression of cancers that are not curable or that have a high risk of recurrence, and (ii) as novel cancer-targeted therapies emerge.

The role of the onco-nephrologist is multifaceted, although one of the most important questions that will need to be addressed in most encounters will be whether continuing cancer-directed therapy is feasible and, if so, how best to support patients through that therapy. Here, we outline 10 tips that address common clinical onco-nephrology encounters in an outpatient setting. The evidence guiding most of these recommendations is largely retrospective, highlighting the need for additional prospective trials in this young but exciting sub-specialty with the potential for rapid growth over the next decade (and beyond).

### Tip 1: Accurate assessment of glomerular filtration rate will better help direct management decisions around type and dose of cancer therapy, and risk of systemic toxicity

Within the realm of oncology, accurate glomerular filtration rate (GFR) estimations are critical as they can influence the pharmacokinetic and pharmacodynamic profiles of cancer therapies [3].

This will influence a patient's treatment eligibility, and will help guide dosing decisions to avoid undertreatment as well as dose-related toxicities, especially with agents that have a narrow therapeutic index [4]. The most accurate assessment of GFR is obtained using exogenous filtration markers, such as iohexol or iothalamate, and monitoring their plasma or urinary clearance to obtain a measured GFR (mGFR). More recently, point-of-care testing with a novel fluorescent GFR tracer, relmapirazin, has also been validated in a small cohort [5]. As mGFR uptake has increased in Europe, the European Kidney Function Consortium recently published a consensus statement on protocol standardization using iohexol mGFR [6]. However, despite the increased uptake, these tests come with practical barriers including availability of the exogenous markers, cost, and time and expertise required [7]. As such, endogenous filtration markers, such as creatinine and cystatin C (Cys), offer a more pragmatic approach.

The limitations of GFR estimating equations (eGFR) based on serum creatinine (sCr) are well recognized. Non-GFR determinants of serum creatinine include diet, muscle mass, proximal tubular secretion, and extra-renal clearance [8]. Moreover, the accuracy of the calculated eGFR relies on appropriate validation of the equation in representative populations [9]. Unfortunately, patients with cancer were not well represented in any of the commonly used eGFR equations such as Cockcroft-Gault (CG), the Modification of Diet Renal Disease Study, and the Chronic Kidney Disease Epidemiology Collaboration (CKD-EPIcr) [10–12]. Furthermore, patients with cancer are often sarcopenic due to the effects of cancer or chemotherapy that result in overestimation of GFR by creatinine. The other common endogenous marker of filtration, Cys—a protein produced by all nucleated cells—is not subject to the same non-GFR determinants as creatinine, although is not without its own limitations [4]. Cystatin C is encoded by a glucocorticoid response gene, associating its production to the exogenous and endogenous presence of glucocorticoids, with higher Cys levels noted in conditions of physiologic stress (obesity, vascular disease), inflammatory conditions, certain cancer types, and with the administration of exogenous steroids, such as in kidney transplant recipients [13–15]. Since it is produced by all nucleated cells, cancer cells are also responsible for Cys production, which can result in underestimation of GFR.

In light of significant limitations with both sCr- and Cys-based equations, the best option may be a combined equation, such as the CKD-EPICr-Cys equation. This question was addressed prospectively in a 1200 patient cohort with solid tumors prior to treatment initiation, in which eGFRs obtained via CG, CKD-EPIcr, and CKD-EPICr-Cys were compared against the results of mGFRs obtained using plasma clearance of a radionuclide [16], Cr-ethylenediaminetetraacetic acid (EDTA), with CKD-EPICr-Cys producing the most accurate results [17]. Similar results were also found in patients with either solid or hematological malignancies, where the CKD-EPICr-Cys equation better reflects mGFR than either eGFRcr or eGFRcys, although, when possible, mGFR may be optimal for clinical decision-making in these populations [18, 19]. Accordingly, both the 2024 KDIGO Chronic Kidney Disease (CKD) guidelines and a recent position statement by the American Society of Onco-Nephrology (ASON) recommend that the combined CKD-EPI Cr-Cys equation be preferentially used in assessing eGFR for patients with cancer [4, 20]. While this recommendation is based on the results of a systematic review, the quality of evidence remains low, indicating that further research is needed.

### Tip 2: Not all elevations in creatinine are indicative of acute kidney injury: cancer-targeted therapy and pseudo-acute kidney injury

**Figure 1:**
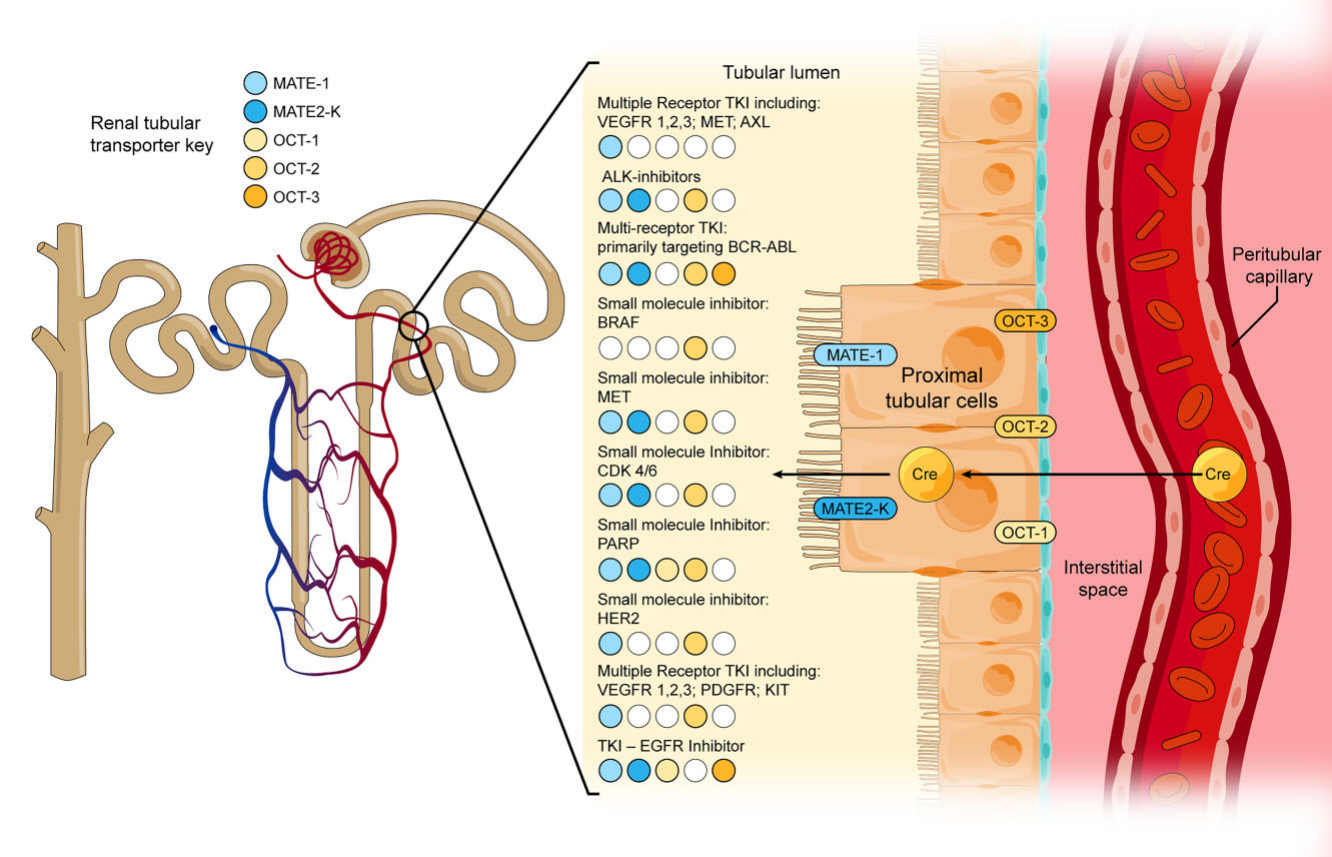
Secreted solutes enter the proximal tubular cells after traversing the interstitium via basolateral transporters, including organic anion transporters and organic cation transporters. Solutes are subsequently secreted into urine via energy-dependent apical transporters. Transporters involved in creatinine secretion, include the OCT-1, OCT-2, and OCT-3 on the basolateral side, and MATE-1 and MATE-2 K on the apical side. The transporters inhibited by different classes of cancer-targeted therapies are depicted in the figure using color-coding; the blocked transporters are denoted by their attributed colors.

Serum creatinine (sCr), a marker of kidney excretory function, is neither a sensitive nor specific marker of acute kidney injury (AKI) [21]. Acute changes in sCr can be a result of multiple processes not associated with kidney injury including: transient physiologic decreases in GFR, increased protein intake or the use of creatine supplements, and the use of medications causing impaired tubular secretion of creatinine [22–24]. The last is especially relevant in the setting of malignancy as multiple cancer-targeted therapies interfere with tubular secretion of creatinine by inhibiting the action of proximal renal tubular transporters such as multidrug and toxin extruder 1 and 2k (MATE-1 and MATE-2k) on the apical surface, organic cation transporter 1, 2, and 3 (OCT-1, OCT-2, OCT-3) on the basolateral surface, and organic anion transporter 2 (OAT2) on the basolateral surface of tubular epithelial cells (Fig. 1) [25]. Further complicating the picture is the ability of targeted therapies to cause renal injury via a variety of mechanisms, rendering the distinction between pseudo-AKI and real AKI critical to decisions around continuing or holding targeted therapies [25, 22]. Table 1 lists the different targeted agents that have been implicated in pseudo-AKI, their mechanism of action, and the renal adverse events [22, 25, 26–49, 16]. The typical clinical course of pseudo-AKI is a rapid rise in creatinine over the first 2 weeks of therapy without other associated markers of renal injury (i.e. proteinuria, active urine sediment), with subsequent sCr plateau, and a rapid return to baseline creatinine with therapy discontinuation [25].

**Table 1:** Cancer-targeted therapies and their association with pseudo-AKI and renal adverse effects [22, 25, 28–49].

Drug name	Drug class	Mechanism of action	Renal tubular transporter inhibited	Potential renal complications
cabozantinib	multiple receptor TKI including: VEGFR 1,2,3; MET; AXL	anti-angiogenic and oncogene directed therapy	MATE-1	§ podocytopathy § TMA
alectinib brigatinib crizotinib ceritinib ensartinib^a^ entrectinib lorlatinib	ALK inhibitors	oncogene directed therapy	OCT-2 MATE-1 MATE-2k	§ ATI § renal cyst formation (crizotinib) § renal arteriolar vacuolization § crescentic GN and AIN (Alectinib) § proteinuria/podocytopathy (lorlatinib)
imatinib	multi-receptor TKI: primarily targeting BCR-ABL	oncogene directed therapy	OCT-2 OCT-3, MATE-1 MATE-2k	§ podocytopathy
vemurafenib	small molecule inhibitor: BRAF	oncogene directed therapy	OCT-2	§ ATI § AIN § proteinuria
capmatinib tepotinib	small molecule inhibitor: MET	oncogene directed therapy	OCT-2 MATE-1 MATE-2k	§ no reports on real AKI to date
palbociclib abemaciclib ribociclib	small molecule inhibitor: CDK 4/6	Cell-cycle arrest	OCT-2 MATE-1 MATE-2k	§ ATI § AIN
olaparib niraparib^b^ rucaparib talazoparib	small molecule inhibitor: PARP	Induces cell death via tumor DNA damage	MATE-1 MATE-2k OCT-1 OCT-2	§ hemodynamic AKI suspected for niraparib
tucatinib lapatinib neratinib	small molecule inhibitor: HER2	oncogene directed therapy	OCT-2 MATE-1	§ no reports of real AKI to date
pazopanib sorafenib sunitinib	multiple receptor TKI including: VEGFR 1,2,3; PDGFR; KIT	anti-angiogenic	MATE-1 OCT-2	§ proteinuria § TMA § pre-eclampsia like syndrome § ATI
gefitinib erlotinib	TKI—EGFR inhibitor	cell-cycle arrest	MATE1 MATE2-K, OCT3 OCT1	§ podocytopathy § endothelial injury

a No data available on Ensartinib.

b Blunted tubular secretion of Cr was not identified in one retrospective study. Abbreviations: VEGFR, vascular endothelial growth factor 1, 2, and 3; MET, mesenchymal epithelial transition factor receptor; AXL, anexelekto; ALK, anaplastic lymphoma kinase; BCR-ABL, breakpoint cluster region-Abelson; BRAF, v-raf murine sarcoma viral oncogene homolog B1; CDK, cyclin-dependent kinase; KIT, stem cell factor receptor; PDGFR, platelet-derived growth factor receptor; PARP, poly(ADP-ribose) polymerase; HER2, human epidermal growth factor receptor 2; ATI, acute tubular injury; TMA, thrombotic microangiopathy; GN, glomerulonephritis; AIN, acute interstitial nephritis.

In cases where sCr is noted to rise, confirming the presence of real AKI can be done by obtaining a follow up Cys and comparing this to the patient's pre-therapy baseline, as Cys is not cleared via tubular secretion [25]. However, serial Cys measurements are not without pitfalls. For one, concomitant corticosteroid use can cause increased serum Cys levels [14]. In addition, serum Cys levels can be lowered irrespective of renal function by cancer treatment, likely due to the release of a Cys cleaving protease, Cathepsin D, from tumor tissue or from the reduction of cancer cells [50]. To date, this phenomenon has been reported for patients on specific tyrosine kinase inhibitors (TKI) [50]. In situations where Cys is unreliable, obtaining a mGFR when available can provide confirmatory testing.

### Tip 3: There are several situations in which measured GFR can alter clinical management

As outlined in the previous two tips, the most commonly used endogenous filtration markers—sCr and Cys—are both associated with significant non-GFR determinants. In line with both the KDIGO 2024 CKD Guidelines and the ASON position statement, we recommend prioritizing a measured GFR, if available in the following instances [4, 20]:

(i) To determine treatment eligibility and drug dosing prior to therapy initiation for patients with:
  (a) Atypical body habitus (i.e. obesity, sarcopenia/malnourished, extremes of height);
  (b) Borderline eGFR for therapy initiation for agents with high potential for nephrotoxicity or narrow therapeutic index
(ii) For serial monitoring in patients starting therapy with agents known to cause pseudo-AKI (see Table 1) as Cys may be affected by tumor response to therapy [50].
(iii) For patients with hematologic malignancies and impaired kidney function undergoing assessment for hematologic stem cell transplantation who may require fludarabine and melphalan conditioning. Post-transplant outcomes, including non-relapse mortality, have been shown to be correlated with mGFR, but not eGFR [51].

### Tip 4: Inhibition of the vascular endothelial growth factor pathway by VEGF inhibitors and tyrosine kinase inhibitors leads to a spectrum of complications from hypertension to renal thrombotic microangiopathy

There are multiple targeted therapies that inhibit the vascular endothelial growth factor (VEGFi) pathway, targeting either soluble VEGF or VEGF receptors. These agents limit tumor growth by inhibiting angiogenesis; they include TKI (i.e. axitinib, cabozantinib, sunitinib), monoclonal antibodies targeting either soluble VEGF or VEGF receptors (i.e. bevacizumab, ramucirumab), and decoy receptors known as VEGF-Traps that impede soluble VEGF from binding to its receptors (Aflibercept) [52]. Renal manifestations of VEGFi include podocyte injury and thrombotic microangiopathy, and are typically dose-dependent, with lower doses less likely to cause complications [48, 52]. Patient presentations span from hypertension in the absence of proteinuria, to nephrotic syndrome with biopsy proven minimal change disease or focal segmental glomerulosclerosis, to systemic thrombotic microangiopathy (TMA), to AKI without systemic features of TMA, but with renal limited TMA (rTMA) that can only be diagnosed with a kidney biopsy [52]. The VEGF pathway is involved in the upregulation of endothelial nitric oxide production, and is an important component of glomerular endothelial-podocyte cross-talk, with podocytes involved in the production of VEGF and endothelial cells expressing VEGF receptors 1 and 2 [48, 53, 54].

**Figure 2:**
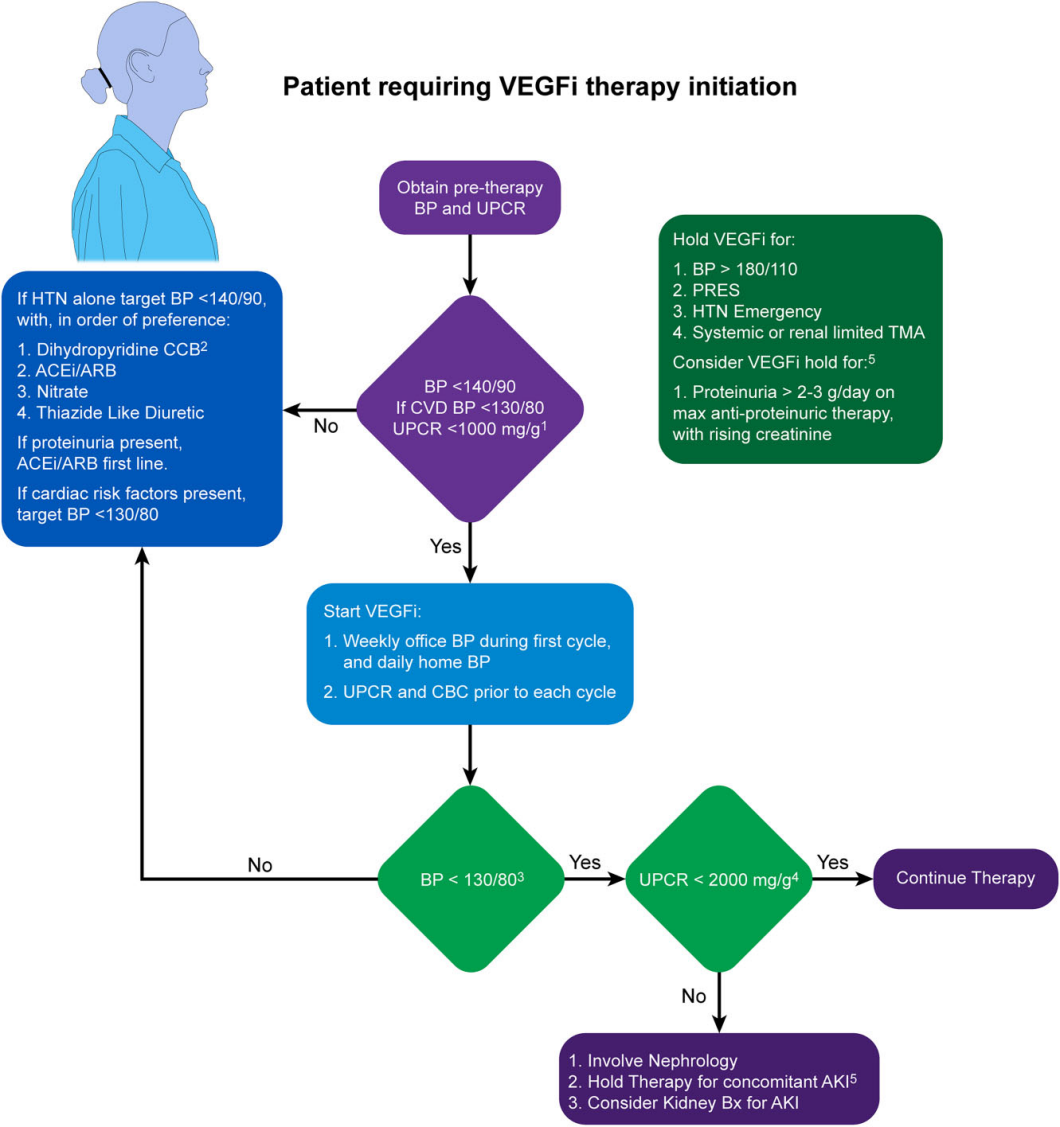
Suggested approach to patients on VEGFi therapy who develop treatment related proteinuria or thrombotic microangiopathy [1]. In the presence of unexplained proteinuria, or active urine sediment, we suggest involving nephrology to consider kidney biopsy prior to VEGFi start to allow treatment of underlying renal disease [2]. Avoid all non-dihydropyridine CCB (e.g. verapamil or diltiazem) with TKI use as they suppress TKI metabolism via cytochrome P450. Treat for stage 1 hypertension (BP 130–139/80–89 mmHg) for patients with cardiac risk factors (i.e. atherosclerotic CVD 10-year risk ≥10%, CKD, diabetes). In the absence of pre-existing cardiac risk factors, can treat for stage 2 hypertension (BP >140/90 mmHg) [3]. With maximum anti-proteinuric therapy on board, and stable creatinine. We suggest confirming presence of >2 g/day proteinuria with 24-hour urine collection [4]. Decisions regarding whether or not to hold/decrease therapy dose to be made collaboratively with oncology. Abbreviations: CVD, cardiovascular disease; BP, blood pressure; UPCR, urine protein to creatinine ratio; HTN, hypertension; CCB, calcium channel blocker; ACEil angiotensin converting enzyme inhibitor; ARB, angiotensin receptor blocker; CBC, complete blood count; PRES, posterior reversible encephalopathy syndrome.

We outline in Fig. 2 our suggested approach to the management of patients on VEGFi [55–59]. Typically, renal complications, including hypertension, TMA, and nephrotic syndrome, resolve with discontinuation of VEGFi [56]. Unfortunately, VEGFi may be the only effective therapy in some patients making discontinuation an unacceptable option. There exist cases in which terminal complement blockade with eculizumab was added for rTMA that was slow to resolve, with clinical benefit [60, 61]. If accessible in a timely manner, functional complement pathway testing may help strengthen the case for the use of terminal complement blockade. Specifically, elevated soluble C5b-9 suggests terminal complement pathway activation, with low AH50, low C3, elevated CBb, and low complement Factor H suggesting increased alternative complement pathway activity. However, as functional complement studies in drug-induced TMA are lacking, the decision to pursue terminal complement blockade is not contingent on serologic evidence of complement activation. As such, this decision can be made on a case-by-case basis, especially when significant systemic features are present or slow to resolve despite the discontinuation of the culprit drug [62]. Complement genetic testing, in addition, could potentially provide insight into predisposing genetic abnormalities in complement proteins, which would also strengthen the case for the use of terminal complement blockade. However, the available biomarkers do not always reliably indicate complement activation and not all gene variants can be considered pathogenic. Drug-induced TMA usually shows a low prevalence of currently identified pathogenic variants [62]. Therefore, recommendations are based on local practice as there is a paucity of evidence to guide best practices in the management of VEGF-i TMA with terminal complement blockade.

Difficult decisions arise in cases where patients develop contraindications to continuing therapy, i.e. nephrotic range proteinuria or TMA, and there are no equally effective alternative oncologic therapies to offer. Using a shared decision-making approach with the patient, oncology and onco-nephrology, a decision can be made to re-initiate VEGFi therapy at a lower dose with weekly monitoring of renal function, proteinuria and blood pressure given its dose-dependent effect [56, 58, 63]. However, the risks of re-initiating therapy need to be acknowledged as part of a risk-benefit discussion. Alternatively, in the presence of terminal complement pathway activation, re-initiating VEGFi therapy with eculizumab therapy may be an option, although there are little data available to support this approach [64].

### Tip 5: Most cases of immune checkpoint inhibitor nephritis are corticosteroid responsive, and confirmatory biopsy should be considered, especially in complex cases

**Figure 3:**
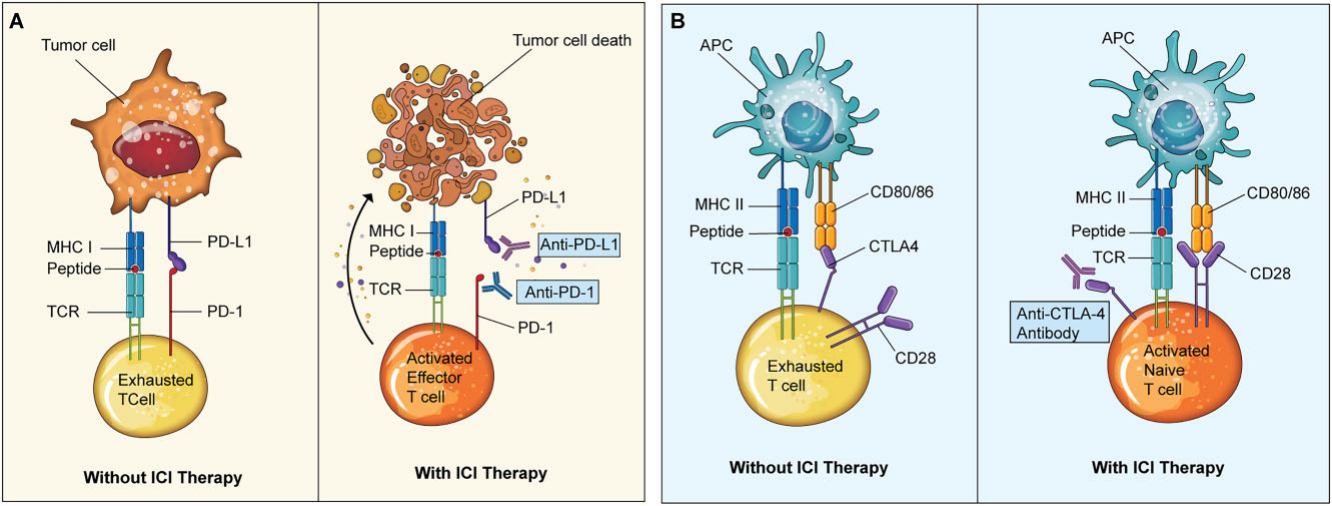
(**A**) Mechanism of PD1/PDL-1 inhibitors. Copied with permission from Barbir *et al.* [65] One mechanism of immune evasion by cancer cells is the expression of PDL-1. Some tumor cells constitutively express PDL-1, PD1’s ligand. Peripherally circulating T cells upregulate the expression of PD1 after they are activated. The binding of PD1 to its ligand, PDL-1, induces T-cell exhaustion, allowing tumor cells to evade cytotoxic T cells. PD1/PDL-1 inhibitors competitively inhibit this mechanism and allow T-cell mediated tumor lysis. MHC, major histocompatibility class; TCR, T-cell receptor. (**B**) Mechanism of CTLA-4 inhibitors. Copied with permission from Barbir *et al.* [65] Within lymphoid tissue, naïve T cells are exposed to tumor associated antigens via antigen-presenting cells. Once the T cell recognizes an antigen as non-self, a second signal, “co-stimulation,” is required for activation. Co-stimulation describes the binding of CD28 on the T cell to CD80/86 on the antigen-presenting cell. Once a T cell is activated, it increases expression of CTLA-4 that binds to CD80/86 with a higher affinity than CD28, thus impeding co-stimulation. Anti-CTLA-4 antibodies bind to CTLA-4, allowing persistent co-stimulation. APC, antigen-presenting cell; MHC, major histocompatibility class; TCR, T cell receptor.

**Figure 4:**
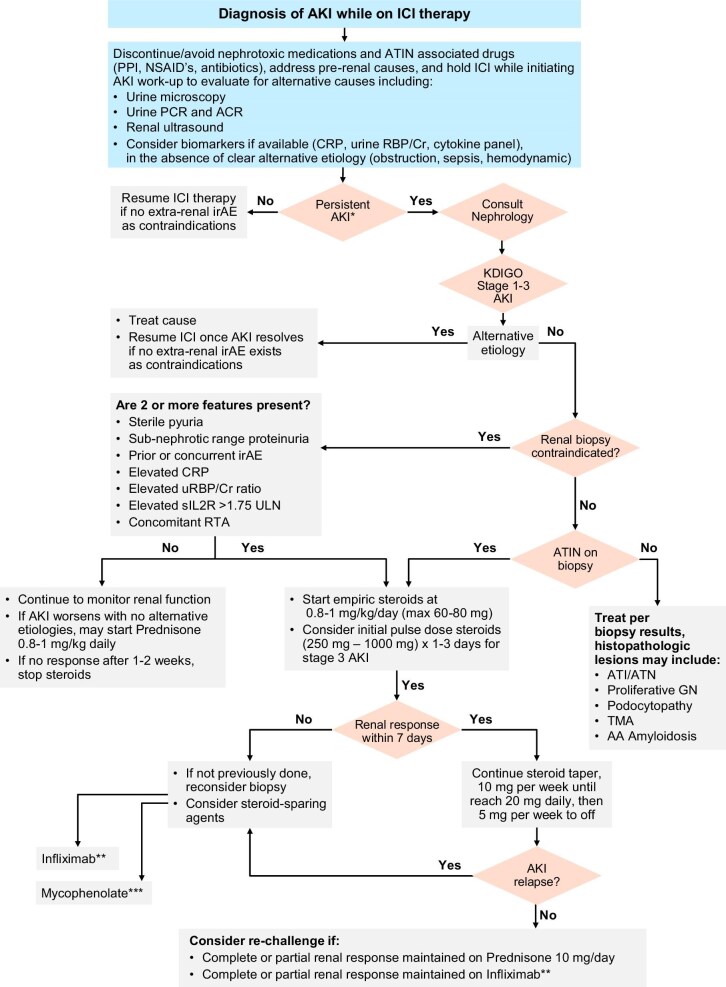
Treatment algorithm for ICI-AKI. Copied with permission from Barbir *et al.* [65] *AKI persisting more than 72 h per KDIGO criteria. **Infliximab is the preferred steroid-sparing agent to trial initially, in the authors’ opinions. ***As mycophenolate is an anti-proliferative agent, it is the authors’ least preferred option for the management of corticosteroid-dependent/refractory ICI-ATIN, as it may affect ICI anti-tumor efficacy, although in resource-limited settings this may be the only accessible option.

Owing to their remarkable efficacy for the treatment of certain tumor types, immune checkpoint inhibitor (ICI) use has expanded significantly since 2011, when the first ICI received Food and Drug Agency (FDA) approval for the treatment of melanoma [65]. There are currently 11 ICIs approved for the treatment of over 20 different cancer types [66]. They are monoclonal antibodies which function by modulating immune response to tumor associated antigens in a non-targeted fashion: they impede T-cell attenuation mechanisms, which subsequently allow ongoing proliferation of activated T cells and T-cell mediated tumor cytotoxicity (Figs 3 and 4). Immunomodulation is facilitated by inhibition of either programmed cell-death 1 or its ligand (PD1, PDL-1) expressed by peripherally circulating T cells, Cytotoxic T-lymphocyte Associated Protein 4 (CTLA-4) expressed by T cells in secondary lymphoid organs, and more recently one agent targeting Lymphocyte Activation Gene 3 (LAG3) received FDA approval [65]. Given their mechanism of action, associated toxicities present as autoimmune complications termed immune related adverse events (irAE), which can affect all organs.

A variety of renal irAEs have been described, ranging from the most common ICI associated AIN (ICI-AIN), to rare complications including glomerulopathies and thrombotic microangiopathy [67]. Based on retrospective data, ICI-AIN is estimated to occur in 1.4%–3% of patients on ICI monotherapy, and up to 5% on ICI dual therapy [68–70]. Our suggested approach to AKI in the cancer patient on ICI therapy is depicted in Fig. 4. Once other causes of AKI have been ruled out, the mainstay of therapy for ICI-AIN consists of withholding ICI therapy and starting corticosteroids, with most cases of ICI-AIN responding to corticosteroid monotherapy at a prednisone equivalent dose of 0.8–1 mg/kg, with a maximum dose of 60–80 mg [70, 71]. Early corticosteroid initiation, within the first 3 days of diagnosis, is associated with increased likelihood of renal recovery [71]. With regards to corticosteroid duration, a tapering course of 4–8 weeks is recommended by a recent ASON position statement [66]. Current clinically available diagnostic markers cannot reliably distinguish ICI-AIN from other etiologies of AKI. Although elevated urine retinol binding protein to creatinine ratio (uRBP/Cr) and serum C-reactive protein (CRP) may suggest ICI-AIN, other causes such as infection and inflammation, as well as other irAEs, must be ruled out [72]. As a result, a kidney biopsy remains the best diagnostic tool, especially for complex cases (e.g. multiple nephrotoxin use) when the risks are not prohibitive, as acute tubular injury may also present with elevated uRBP/Cr and other extra-renal irAEs can increase serum CRP levels. Other clinically available potential diagnostic tools of ICI-AIN such as sIL-2R and PET-CT Kidney SUV scan have not been appropriately compared to any patient with AKI on ICI therapy (only to non-AKI controls on ICI or AKI patients not on ICI therapy) [73, 74]. In addition, other factors can affect both sIL-2R levels (e.g. other extra-renal irAEs, inflammatory state) and PET kidney SUV (e.g. any significant AKI, time to imaging after FDG injection, burden of metastatic disease taking up FDG, dose of FDG injected, and fasting state) [65, 75, 76]. Therefore, additional studies with appropriate controls are needed.

### Tip 6: Infectious prophylaxis can be tailored to the individual patient

Immunosuppressive drugs come with an associated risk of opportunistic infections. In the case of a prolonged course of corticosteroid therapy, prophylaxis is required for *Pneumocystis jiroveci* pneumonia (PJP). The National Comprehensive Cancer Network guideline for the management of ICI-related toxicities recommends initiating sulfamethoxazole trimethoprim (Bactrim) first line for patients who are expected to require corticosteroid therapy for 4 weeks or more, with a prednisone equivalent dose of 20 mg or higher [77]. However, they suggest that starting PJP prophylaxis at 2 weeks is reasonable for patients who are expected to remain corticosteroid dependent [77]. For patients with a sulfa allergy, they recommend IV pentamidine as the preferred alternative over dapsone due to the risk of hemolytic anemia, and over atovaquone due to the risk of diarrhea (especially relevant for patients with concurrent ICI-colitis) [28].

In our practice, given the existence of two recent meta-analyses demonstrating a strong association between AIN-associated drug use (bactrim, proton-pump inhibitors, non-steroidal anti-inflammatory drugs) and the development of ICI-AIN, we avoid the use of bactrim for PJP prophylaxis in patients with ICI-AIN [78, 79]. We favor inhaled pentamidine when feasible, with judicious use of either dapsone or atovaquone on a case-by-case basis when pentamidine is not available. We typically start PJP prophylaxis within 2 weeks of the start of glucocorticoids, anticipating a course of therapy that will exceed 4 weeks.

### Tip 7: Steroid-sparing therapies are an option for corticosteroid-dependent or -refractory ICI-AIN

While most patients with ICI-AIN will respond to corticosteroid monotherapy, there exist a small subset of patients that are corticosteroid dependent or refractory [80, 81]. Corticosteroid-dependent patients require higher doses of steroids (i.e. in excess of 20 mg daily) to avoid relapse of ICI-AIN. Recent data suggest that higher peak corticosteroid doses used for the treatment of ICI toxicity in patients with cancer (in excess of 0.5 mg/kg) are associated with adverse outcomes, with decreased progression free survival and overall survival, while the same association was not noted for higher cumulative corticosteroid doses [82]. Steroid-sparing therapies offer an opportunity to decrease peak corticosteroid doses in biopsy proven corticosteroid-dependent ICI-AIN, and to treat corticosteroid-resistant AIN.

The available retrospective data on steroid-sparing therapy for ICI-AIN, although limited to case series and case reports, currently support the use of infliximab, a tumor necrosis factor alpha inhibitor. Infliximab is dosed monthly at 5 mg/kg, with only 1–3 doses typically required. As 1 to 2 weeks is required for clinical response, we taper corticosteroids more aggressively 2 weeks after the first dose [80]. Contraindications to Infliximab use are listed in Box 1. For those patients who are not candidates for infliximab therapy, reported alternative steroid-sparing therapies include mycophenolate mofetil (MMF) and tocilizumab, although we favor tocilizumab as MMF is an anti-proliferative agent [65].

Box 1: **Contraindications to Infliximab Therapy Initiation**

- Latent or Active Tuberculosis (TB). Latent TB must be ruled out prior to therapy initiation.
- Active infections including Hepatitis B, Hepatitis C, and HIV
- New York Heart Association Class III or IV Heart Failure
- History of severe hypersensitivity reactions to Infliximab or any murine proteins
- Pre-existing transaminitis unless it is secondary to ICI associated hepatitis, in which case consultation with gastroenterology prior to therapy start is needed

### Tip 8: Kidney transplant recipients stand to benefit from ICI therapy

Kidney transplant recipients (KTR) comprise a unique population with regards to ICI therapy given the need for long term immunosuppression to preserve allograft tolerance, and concerns that ICI therapy may (i) be less effective with maintenance immunosuppression present, and (ii) may trigger rejection given the non-targeted mechanism of action of ICIs [83]. However, novel and effective oncologic therapies are urgently needed for a population at increased risk of malignancy compared to the general population, and for whom malignancy represents one of the leading causes of death with a functional allograft post-transplant [84, 85]. Early experience with ICI therapy in KTR involved significant minimization or discontinuation of maintenance immunosuppression, with high allograft rejection rates on the order of 40%–50%, with half of those patients subsequently losing their allografts [86, 87]. Of note, the allograft biopsy rate in the available retrospective studies is ∼50%, further limiting the quality of the retrospective data [86]. Encouragingly, objective response to therapy, especially for certain common tumor groups such as cutaneous squamous cell carcinoma (cSCC), were noted to be comparable to those seen in the general population, despite the use of maintenance immunosuppression [83, 87]. Moreover, three recent prospective studies, listed in Table 2, that either maintained baseline immunosuppression with ICI initiation, used mammalian target of rapamycin inhibition (mTORi) with a dynamic mini-steroid pulse or a combination of low dose tacrolimus and prednisone, revealed rejection rates on the order of 0%–38% [88–90]. Taken together, recent evidence suggests that KTR with specific tumor types, such as cSCC, stand to benefit significantly from ICI therapy with optimization of maintenance immunosuppression. Additional work is needed to determine prognostic markers to help guide who would benefit most from ICI initiation, as well as to determine optimal immunosuppression regimens [66]. In the interim, patients would benefit from a shared decision-making approach, involving their oncologist, and transplant and onco-nephrology teams, to understand the potential benefits of ICI therapy in the context of the existing risks.

**Table 2:** The three published prospective trials to date on the use of ICI therapy in KTRs. Adapted from Barbir *et al.* [83]^.^

Study title	ICIs in KTR [88]	CONTRAC-1 [113]	Nivolumab + tacrolimus + prednisone ± ipilimumab for KTR with advanced cutaneous cancers [114]
Authors, year	Carroll *et al.*, 2022	Hanna *et al.*, 2024	Schenk *et al.*, 2024
Patient number	17	12	8
Tumor group	Any advanced cancer otherwise meeting ICI indication	advanced cSCC	advanced skin cancers
ICI type	16 patients on anti-PD1 therapy 1 patient on anti-PDL1 therapy	cemiplimab (Anti-PD1)	Initial Nivolumab (Anti-PD1) in 8/8 patients Transition to nivolumab + ipilimumab (anti-CTLA4) in 6/8 patients
Maintenance immunosuppression	Maintain prior baseline maintenance immunosuppression	mTORi and dynamic prednisone taper	Tacrolimus (trough 2–5 ng/ml) and Prednisone 5 mg daily
Rejection	2/17, 11.7%	0/12, 0%	3/8, 37.5%
Allograft biopsy findings	2 cases of ACR	N/A	1 case of ACR, mixed rejection (ACR + ABMR) × 2
Extra-renal immune related adverse events	1/17, colitis	1/12, colitis	2/8, arthralgia, maculopapular rash
Objective response rate (%)	53	45	25

Abbreviations: ACR, acute cellular rejection; mTORi, Mammalian Target of Rapamycin Inhibitor; ABMR, antibody mediated rejection. Objective response rate is defined as the proportion of patients with a complete response or partial response to treatment according to Response Evaluation Criteria in Solid Tumors.

### Tip 9: Multiple myeloma and AL amyloidosis patients with end stage kidney disease would benefit from early referral for kidney transplant evaluation

The prognosis of plasma cell disorders, comprising the spectrum of plasma cell malignancy such as multiple myeloma (MM), to monoclonal gammopathy of renal significance, such as light chain amyloidosis (AL), has dramatically improved over the last two decades due to novel anti-myeloma and plasma cell directed therapies [91]. Patients with MM eligible for autologous stem cell transplant have an OS >8 years, compared to <1 year in the 1980s [92–94]. Similarly, for patients with AL amyloidosis without cardiac involvement, the 5-year median survival has increased to 71% in the recent era compared to 19% in the 1980s [95].

In the case of MM, dialysis-dependent patients have significantly worse outcomes, with a standardized mortality ratio 22 times higher than MM patients not on dialysis [96]. Patients with AL amyloidosis on dialysis face restricted treatment options, have a poorer quality of life compared to those not on dialysis, and have a reported median OS of 39 months [94, 97, 98]. There are potential survival and quality of life benefits to pursuing kidney transplantation in this population. However, transplant referrals for patients with MM and AL Amyloidosis are infrequent, in part due to the absence of definitive society guidelines and uncertainty surrounding patient and allograft outcomes [94, 99]. The other unique feature in this population is the absence of a pre-defined waiting time, as both MM and AL Amyloidosis have a high risk of recurrence, and avoiding too long a waiting time during a period of hematologic remission likely confers benefit [91].

Recent data on outcomes post kidney transplant for MM and AL Amyloidosis patients is encouraging. A United States Organ Procurement and Transplantation Network/National United Network for Organ Sharing (OPTN/UNOS) database analysis from 1988 to 2019 identified 218 KTR with MM with a median overall survival of 8.4 years [100]. While a survival difference was identified in KTR with MM, this was lost when comparing groups older than 50 years of age. Similarly, an international multi-center study reviewing the outcomes of 237 KTR with AL amyloidosis revealed a median overall survival of 8.6 years, and a 5-year OS comparable to KTR over the age of 65 [101]. In light of these outcomes, efforts are currently underway to create a unified approach to the evaluation and selection of these patients.

### Tip 10: Not all monoclonal gammopathies of renal significance will have a detectable clone by standard diagnostic tests

Monoclonal immunoglobulins can cause renal toxicity via multiple mechanisms [102]. The concept of monoclonal gammopathy of renal significance (MGRS) is now well established although the term was coined just over a decade ago, in 2012, by Leung, Bridoux *et al.* on behalf of the International Kidney and Monoclonal Gammopathy Research Group [103]. The creation of a new diagnostic category helped: (i) highlight the capacity of monoclonal proteins to cause renal injury due to their physicochemical properties rather than due to the clonal burden, and (ii) justify the use of clone-directed therapy for a pre-malignant condition from a hematologic standpoint [104–107]. However, determining the appropriate clone-directed therapy (i.e. treatment targeting a plasmacytic vs lymphocytic clone) becomes more challenging when a pathologic clone is not detectable. Of the MGRS lesions, nearly all patients with AL amyloidosis and Randall-type monoclonal immunoglobulin deposition disease will have a detectable pathological clone, whereas clones may be difficult to identify in other diseases [106]. The latter may change as our diagnostic tools improve. Pathologic clone detection is typically achieved via bone marrow or peripheral blood cytologic and flow cytometry analysis. In a recent study, Javaugue *et al.* were able to identify a pathologic clone in all 16 patients with biopsy proven MGRS that were tested. A significant subset of these patients did not have a detectable clone by bone marrow biopsy immunohistochemical analysis/flow cytometry or peripheral blood flow cytometry, and two of the cases did not even have a detectable circulating immunoglobulin [108]. Javaugue *et al.* achieved a much higher test sensitivity using a high-throughput sequencing assay from bone marrow mRNA encoding immunoglobulins (RACE-RepSeq) [108].

Until clinical integration of more sensitive testing is feasible, treatment decisions will continue to be made in the absence of a pathologic clone in a subset of cases. Proliferative glomerulonephritis with monoclonal immunoglobulin deposits (PGNMID) is well recognized to have a low rate of detectable pathologic clones, with a clone identified in only ∼30% of reported cases, and only 20%–30% have a detectable circulating monoclonal immunoglobulin, which makes it difficult to determine hematologic response to clone-directed therapy [106, 109]. Given an even distribution of pathologic clones in PGNMID cases with detectable clones, ∼50% lymphocytic and 50% plasmacytic, a reasonable treatment approach would be to start by targeting a lymphocytic clone with anti-CD20 therapy, and switch to plasma cell directed therapy after 2–3 cycles in the absence of renal response to therapy [110, 111]. However, a recent open-label prospective trial including 10 patients with PGNMID all treated with plasma cell directed therapy with daratumumab, an anti-CD38 monoclonal antibody, induced a complete response in six patients and partial response in four patients within 1 year [112]. The latter suggests that first line daratumumab, when accessible, may be a better strategy, with the exception of patients with IgM deposits as these are typically produced by CD20 positive cells [109].

## SUMMARY

Oncologic therapies are evolving at a rapid pace. Onco-nephrologists offer valuable expertise with regards to timely diagnosis of renal toxicities, relying on kidney biopsy and/or novel biomarkers depending on the clinical circumstances. Moreover, onco-nephrologists work closely with oncologists to allow safe continuation or prompt re-initiation of cancer therapy, when possible. One of the challenges faced by onco-nephrology is the lack of high quality, prospective data to help guide clinical decision-making. This issue is due in part to the relative novelty of the field with disease definitions in evolution, and standardized renal-specific outcomes lacking. In recognition of this limitation, international societies, such as the International Kidney and Myeloma Working Group, are enabling multi-center data sharing to better power future studies. The opportunity exists for nephrologists and hematologists/oncologists to collaborate on the establishment of renal-specific endpoints in large, prospective trials as well. Last, disease specific or drug-specific international registries offer an alternative to formally study off-target renal side-effects and their management.
